# A Range-Aware Attention Framework for Meteorological Visibility Estimation

**DOI:** 10.3390/s26061893

**Published:** 2026-03-17

**Authors:** Wai Lun Lo, Kwok Wai Wong, Richard Tai Chiu Hsung, Henry Shu Hung Chung, Hong Fu, Harris Sik Ho Tsang, Tony Yulin Zhu

**Affiliations:** 1 Department of Computer Science, Hong Kong Chu Hai College, Hong Kong, China; edmondwong@chuhai.edu.hk (K.W.W.); richardhsung@chuhai.edu.hk (R.T.C.H.); harristsang@chuhai.edu.hk (H.S.H.T.); ylzhu@chuhai.edu.hk (T.Y.Z.); 2 Department of Electrical Engineering, City University of Hong Kong, Hong Kong, China; eeshc@cityu.edu.hk; 3 Department of Mathematics and Information Technology, Education University of Hong Kong, Hong Kong, China; hfu@eduhk.hk

**Keywords:** artificial neural network, deep learning, hybrid architecture, meteorological visibility estimation

## Abstract

Accurate meteorological visibility estimation is critical to the safety and reliability of transportation and environmental monitoring systems. Despite the prevalence of deep learning, models often struggle with the non-linear visual degradation caused by varying atmospheric conditions and a scarcity of instrument-calibrated datasets. This study makes two primary contributions. First, we introduce the Hong Kong Chu Hai College Visibility Dataset (HKCHC-VD) comprising 11,148 high-resolution images paired with precise visibility measurements from a Biral SWS-100 sensor. Second, we propose a Range-Aware Attention Framework (RAT-Attn), an adaptive attention mechanism that translates classical range-specific atmospheric modeling into differentiable deep learning operations. This is a domain-specific architectural optimization that integrates a dual-backbone architecture (CNN and Vision Transformer) with a learnable threshold mechanism. This design enables the model to dynamically prioritize spatial and channel-wise features based on estimated visibility intervals, specifically targeting the non-linear visual degradation unique to fog and haze. Experimental results demonstrate that our proposed approach outperforms existing baselines, including VisNet and landmark ANN-based methods. The ResNet + ViT (spatial-threshold) variant achieves the most balanced performance, recording a Mean Squared Error (MSE) of 5.87 km^2^, a Mean Absolute Error (MAE) of 1.65 km, and a classification accuracy of 87.07%. In critical low-visibility conditions (0 to 10 km), the framework reduces regression error by over 75% compared to the baselines. These results confirm that range-aware adaptive feature fusion is essential for robust meteorological estimation in real-world environments.

## 1. Introduction

Meteorological visibility is an important indicator of air quality, and it represents the maximum distance that an object can be recognized against a bright background. Meteorological visibility also represents the transparency of the surrounding air, affecting the safety of road, flight and maritime traffic. It can be used as an indicator for air pollution and weather conditions. Visibility measurement can be performed using sophisticated visibility meters or by image analysis methods. The Meteorological Optical Range (MOR) is a measure of atmospheric transparency which can be measured by sophisticated visibility meters [1] or a well-trained meteorological expert can estimate the MOR by observing the largest visible distance in a scene. However, this method is dependent on the judgment and perception of the observer [1]. For the manual method, Liu [2] compared visibilities obtained by instrumental measurement and visual observation. The study found that the accuracy of manual methods is affected by the number of landmark objects in the scene. A method for measuring visibility distance by imaging a linear grating was proposed in [3], which is based on contrast measurement in a digital image. However, such measurements are often compromised by fluctuating lighting conditions, suspended particles, and the environmental context [4]. Physically, visibility is most commonly measured via forward or backward scattering [5]. The forward scattering method is widely applied in visibility meters because of its lower cost and reasonable accuracy within the design range. However, high precision forward scattering visibility meters [6] remain very expensive and require a well-trained technical expert for calibration and installation.

To address the limitations of hand-crafted features and calibration constraints inherent in physical models, the field has pivoted toward deep learning-based approaches that learn atmospheric representations directly from data. Unlike early artificial neural networks (ANNs) that relied on pre-defined edge detection or contrast features [7,8,9,10,11,12,13], modern Convolutional Neural Networks (CNNs) automatically extract hierarchical feature maps that are robust to varying illumination. Subsequent studies have leveraged deeper backbones, such as ResNet [14] to improve regression accuracy by capturing high-level semantic dependencies in foggy scenes. Similarly, pre-trained CNNs such as AlexNet, GoogLeNet, and ResNet18 have been proposed to classify diverse weather conditions [15]. Recent literature highlights the versatility of these approaches. Li proposed a visibility evaluation based on webcam weather images using deep learning features in [16]. This evolution continued with the development of specialized regression frameworks such as VisNet [17] and TVRNet [18], the latter of which introduced a deeply integrated CNN architecture to fuse global scattering features with local contrast information.

A visibility estimation method using a hybrid neural network with localized image entropy and image-based features as inputs was proposed in [19], while multi-model deep fusion networks have also been proposed to handle smaller training datasets [20,21,22,23]. Further research introduced Recurrent Neural Networks (RNNs) to process temporal dependencies in video sequences and hybrid networks that incorporate image entropy [24,25,26] or Fourier transforms to capture frequency-domain characteristics [27].

Current state-of-the-art methods for visibility estimation continue to push the boundaries of accuracy [28,29]. Specifically, ref. [29] introduced a method for estimating visibility using 3D point clouds and road network maps, while machine learning-based visibility estimation has been shown to provide safer navigation [30] in the Strait of Istanbul. Innovative end-to-end CNN frameworks have also been proposed, such as the DMRVisNet [31] and STCN-Net approach [32]. Additionally, VISOR-NET [33] was proposed for visibility estimation by using ordinal information and relative relations between images, and [34] employed a machine learning algorithm to estimate the visibility based on particulate matter and weather data. While Pavlove [35] studied different deep learning methods for airport visibility, ref. [36] proposed a Deep Quantified Visibility Estimation Network consisting of Transmission, Depth, and the Extinction Coefficient Estimation Modules. Recent specialized efforts include Xiao’s [37] multi-scale fusion network for highway visibility and methods tailored for nighttime estimation [38]. Finally, ref. [39] provides a comprehensive overview of atmospheric visibility estimation using deep learning-based methods.

Simultaneously, applying large vision-language models (VLMs) to visual tasks is becoming an increasingly popular trend. To preliminarily evaluate the performance of state-of-the-art commercial VLMs, we randomly selected images from different visibility ranges within our dataset and employed a prompting strategy (“Estimate the meteorological visibility in kilometers for this image. Provide a single numerical value or a narrow range.”) for testing. However, as shown in Table 1, even state-of-the-art commercial VLMs perform poorly in meteorological visibility estimation. Their predictions are consistently underestimated, particularly in mid-range visibility conditions (10–30 km), where errors can deviate by factors of 2–5 times the actual values. This performance gap indicates that current VLMs are not optimized for the subtle atmospheric scattering and contrast degradation patterns critical for accuracy. This failure underscores the critical need for domain-specific architectural optimizations.

In this paper, we introduce the Range-Aware Attention Framework (RAT-Attn), which integrates a learnable threshold mechanism with range-aware attention modules. This design enables the model to adaptively prioritize visual features based on the estimated visibility interval, overcoming the constraints of traditional general-purpose architectures. The remainder of this paper is organized as follows: Section 2 details the proposed range-aware attention framework, including the dual-backbone architecture and feature extraction, the RAT-Attn mechanism, the multi-head prediction architecture, as well as the multi-task loss function. Section 3 provides a comprehensive analysis of the estimation and classification results. Section 4 offers a discussion of the methodology’s implications, and Section 5 summarizes the conclusions of this work.

## 2. Proposed Range-Aware Attention Framework

Traditional methods for object detection or image classification typically rely on a single CNN backbone for feature extraction [40]. However, in the context of visibility estimation, while such approaches may be sufficient for categorizing visibility into broad classes (e.g., low, medium, high), they often struggle to provide accurate continuous visibility estimates. Our proposed architecture integrates local texture details from a CNN with range-aware attention to strongly suppress irrelevant regions or noisy channels and combines them with the global atmospheric context captured by a ViT feature extraction, thereby enabling adaptive feature processing tailored to different visibility conditions.

### 2.1. Architecture Overview

**Figure 1:**
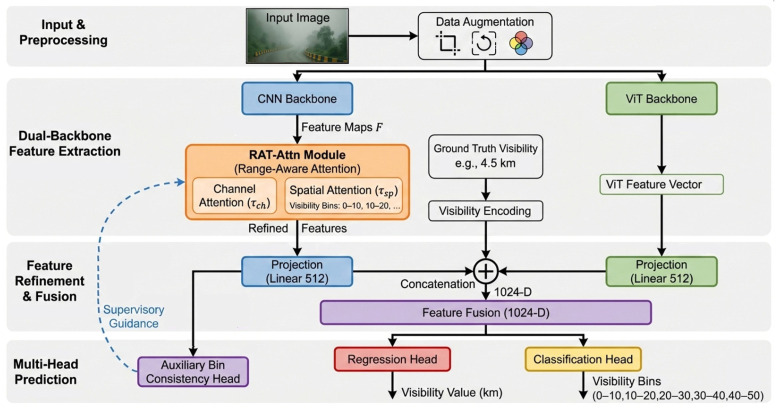
End-to-end architecture of the proposed Range-aware Attention Framework (RAT-Attn).

As illustrated in Figure 1, the end-to-end framework is structurally divided into four primary stages: (1) Input & Preprocessing, (2) Dual-Backbone Feature Extraction, (3) Feature Refinement & Fusion, and (4) Multi-Head Prediction. After initial data augmentation to enhance robustness against environmental variations, images are processed by a dual-backbone feature extraction module. Guided by encoded visibility ground truth (0–50 km range) data, the output feature vectors from each pathway of the dual-backbone are then concatenated, fusing local texture details with global atmospheric context. This rich feature set feeds into our multi-task prediction heads: the Regression head, the Classification Head, and the Auxiliary Bin Consistency Head. The regression head outputs the continuous visibility distance predictions, and the classification head estimates bin probabilities, while the auxiliary bin consistency head provides additional supervision directly from pooled CNN features to ensure prediction alignment across visibility ranges.

### 2.2. Input and Preprocessing

The network accepts raw meteorological images as input. To ensure robustness against lighting variations, sensor noise, and varying camera angles, the input images undergo a data augmentation pipeline during training. This includes random cropping, rotational shifts, and color jittering. Also, all input images are resized to 224 × 224. These augmentations prevent the model from overfitting to specific static scenes and force the network to learn generalized atmospheric scattering cues.

### 2.3. Dual Backbone Architecture and Feature Extraction

The dual-backbone architecture leverages the distinct strengths of convolutional and transformer-based processing to capture both localized details and global contextual relationships essential for accurate visibility assessment. The dual pathways process images in parallel, with each backbone specializing in different aspects of visibility estimation. The CNN backbone processes the input image to extract high-resolution spatial feature maps *F.* In our experiment, the ResNeXt is employed for the CNN pathway. This pathway is designed to be highly sensitive to local textural changes and contrast degradation caused by haze. Within this pathway, features undergo transformation through our proposed Range-Aware Learnable Threshold Attention module, which implements visibility bin-specific processing across five visibility bin intervals: 0–10 km, 10–20 km, 20–30 km, 30–40 km, and 40–50 km. This module dynamically applies learnable thresholds to both channel and spatial attention mechanisms, providing advantages in variable conditions compared to traditional attention approaches [41]. Thus, it provides critical capabilities including texture analysis for atmospheric particles, edge preservation for object boundaries, and identification of visibility indicators through sensitivity to local contrast.

While the ViT backbone extracts a robust global representation (ViT Feature Vector) through patch-based self-attention mechanisms, it can model long-range dependencies across the scene, capture atmospheric gradients, and understand spatial relationships between terrain, horizon, and sky regions. It is excellent for capturing these long-range dependencies and the overall atmospheric context of the scene.

### 2.4. Range-Aware Learnable Threshold Attention System

The core innovation of this work is the Range-Aware Learnable Threshold Attention module, which specifically addresses the spatial non-uniformity inherent in atmospheric phenomena and dynamically refines the CNN feature maps. Unlike standard attention mechanisms, RAT-Attn introduces a learnable threshold (τ) to handle the non-linear nature of light scattering. This complexity is justified because different visibility ranges require the model to focus on distinct image components: nearby objects provide critical cues for low-visibility conditions, whereas distant landmarks are essential for high-visibility estimation. By discretizing visibility into range-specific bins, the model learns thresholds that effectively filter atmospheric noise in obscured scenes while preserving salient long-range features in clear conditions.

**Figure 2:**
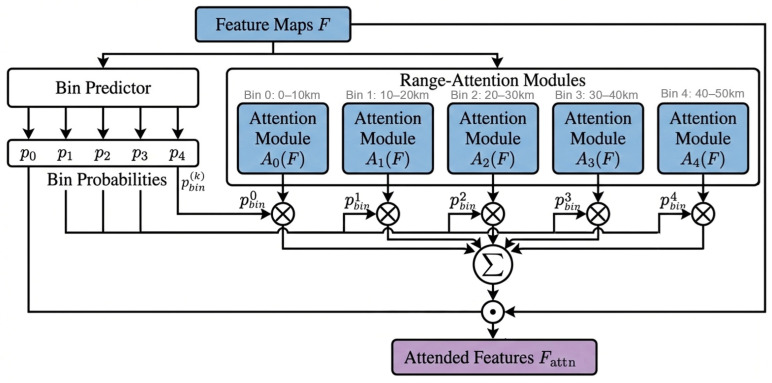
Block Diagram of the Range-Aware Learnable Threshold Attention.

As detailed in Figure 2, the Range-Aware Learnable Threshold Attention module applies bin-specific attention conditioned on visibility ranges partitioned into five intervals: [0–10 km), [10–20 km), [20–30 km), [30–40 km), and [40–50 km]. The bin probabilities $p_{bin}$ are derived from the CNN feature maps (*F*) via a Multi-Layer Perceptron (*MLP*) and Global Average Pooling (*GAP*):

(1) $$p_{bin}=\mathrm{softmax}\left(MLP\left(GlobalAvgPool\left(F\right)\right)\right)$$

Each bin incorporates dedicated channel and spatial attention components with learnable thresholds. The channel attention mechanism mainly consists of a base attention module ($ChAttn_{bs}$) and a threshold gate ($ChThr_{gate}$). The *k*-th bin-specific channel attention block is defined as:

(2) $$ChAttn^{\left(k\right)}\left(F\right)=F⊙ChAttn_{bs}⊙ChThr_{gate}$$

where

(3) $$ChAttn_{bs}=\sigma \left(MLP\left(AvgPool\left(F\right)+MaxPool\left(F\right)\right)\right)$$

(4) $$ChThr_{gate}=\sigma \left(\alpha \cdot \left(ChAttn_{bs}-\tau_{c}^{\left(k\right)}\right)\right)$$

In these equations, *AvgPool*(*F*) and *MaxPool*(*F*) denote global average and max pooling operations, respectively. The *MLP* utilizes a reduction ratio of 16, $\tau_{c}^{\left(k\right)}$ represents the learnable channel threshold for bin *k*, while α = 10 acts as a sharpness factor to ensure a differentiable threshold approximation, and σ denotes the sigmoid activation function. Similarly, the spatial attention mechanism targets relevant spatial regions while suppressing background textures. It utilizes a 7 × 7 convolutional layer on concatenated global average and max-pooled features, with bin-specific learnable thresholds $\tau_{s}^{\left(k\right)}$. The *k*-th bin spatial attention block is defined as:

(5) $$SpAttn^{\left(k\right)}\left(F\right)=F⊙SpAttn_{bs}⊙SpThr_{gate}$$

where

(6) $$SpAttn_{bs}=\sigma \left(f_{7\times 7}\left(\left[Avg\left(F\right);Max\left(F\right)\right]\right)\right)$$

(7) $$SpThr_{gate}=\sigma \left(\alpha \cdot \left(SpAttn_{bs}-\tau_{s}^{\left(k\right)}\right)\right)$$

Here, [*Avg*(*F*) and *Max*(*F*)] represents the concatenation of feature maps pooled across the channel dimension, $f_{7\times 7}$ denotes a convolution with a kernel size of 7 × 7 (padding = 3), and $\tau_{s}^{\left(k\right)}$ is the learnable spatial threshold for bin *k*. Both components incorporate a sharpness factor α = 10 for differentiable threshold approximation. The final outputs of the bin-specific blocks are combined through a probability-weighted summation to produce refined feature maps:

(8) $$F_{attn}=\sum_{k=1}^{K}p_{bin}^{\left(k\right)}{\cdot \;\mathrm{SpAttn}}^{\left(k\right)}\left({\mathrm{ChAttn}}^{\left(k\right)}\left(F_{CNN}\right)\right)$$

These attended CNN features are input to the Feature Refinement & Fusion stage for further processing.

### 2.5. Feature Refinement and Fusion

Using the provided encoded visibility ground truth data, the attended CNN features are subsequently projected to a 512-dimensional space and concatenated with the Vision Transformer (ViT) features, resulting in a 1024-dimensional feature vector that serves as the input for the multi-head prediction stage.

### 2.6. Multi-Head Prediction Architecture

The feature fusion process strategically combines complementary representations from both backbones into a unified feature space through the concatenation the projected CNN and ViT features, forming a 1024-dimensional vector. To address the inherent ambiguity and spatial non-uniformity of atmospheric scattering, we feed this representation into three specialized prediction heads. This multi-task structure provides mutual regularization that prevents the model from overfitting to localized artifacts like rain streaks or sensor noise.

**Figure 3:**
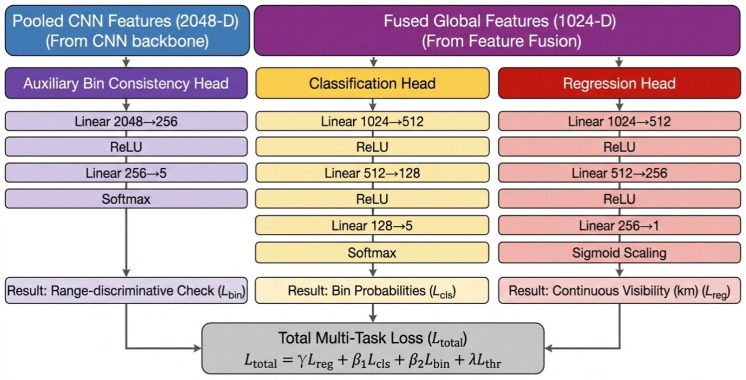
Hierarchical Multi-Task Prediction Heads & Loss Function.

The architecture of the multi-task prediction heads is illustrated in Figure 3. The Regression Head processes fused features through a three-weight-layer MLP (Linear (1024, 512) → ReLU → Linear (512, 256) → ReLU → Linear (256, 1)). The hierarchical reduction in dimensionality (1024 → 512 → 256) ensures that the model distills high-level semantic features before performing the final regression. A sigmoid scaling layer constrains the output to the physically plausible 0–50 km range.

The Classification Head (Linear (1024, 512) → ReLU → Linear (512, 128) → ReLU → Linear (128, 5)) categorizes scenes into five meteorological bins [0–10, 10–20, 20–30, 30–40, 40–50 km]. It forces the network to learn discrete ordinal relationships between visibility intervals while preventing overfitting to the specific textures of a single bin.

The Auxiliary Bin Consistency Head acts as a mid-stream supervisor processing pooled CNN features directly (Linear (2048, 256) → ReLU → Linear (256, 5)). It ensures that range-discriminative features remain intact within the CNN backbone before they are fused with the global ViT context. This independent supervision path is critical for maintaining physical consistency. It prevents attention drift that can occur when global context (like sky color) overrides local visibility cues (such as object contrast).

### 2.7. Multi-Task Loss Function

The composite loss function $L_{total}$ coordinates all prediction heads through balanced multi-task optimization, combining four weighted components: regression loss $L_{reg}$, classification loss $L_{cls}$, auxiliary classification loss $L_{bin}$, and threshold regularization $L_{thr}$. The total loss $L_{total}$ is defined as:

(9) $$L_{total}=\gamma L_{reg}+\beta_{1}L_{cls}+\beta_{2}L_{bin}+\lambda L_{thr}$$

The parameter γ prioritizes the primary task of continuous distance regression. The $\beta_{1}$ and $\beta_{2}$ weights are determined through an empirical sensitivity analysis. It should be set large enough to regularize the feature space while preventing classification gradients from dominating the regression gradients, thus avoiding training instability. The parameter λ is a small regularization penalty to ensure that the learnable thresholds *τ* do not deviate into physically impossible values.

The primary objective is the accurate estimation of the continuous visibility distance. The regression loss $L_{reg}$ combines Mean Absolute Error (MAE) and Mean Squared Error (MSE) terms, which are defined in Equations (15) and (16), to enhance distance estimation precision while maintaining robustness to outliers:

(10) $$L_{reg}=\frac{1}{N}\sum_{i=1}^{N}\left(∥{\hat{v}}_{i}-v_{i}{∥}_{1}+{\left({\hat{v}}_{i}-v_{i}\right)}^{2}\right)$$

where *N* is the batch size, $v_{i}$ is the ground-truth visibility value, and ${\hat{v}}_{i}$ is the model’s predicted value for the *i*-th sample. To provide categorical guidance and improve convergence, we incorporate two Cross-Entropy (CE) loss components. $L_{cls}$ supervises the final classification head, while $L_{bin}$ acts as an auxiliary supervisor for the CNN backbone. This ensures that the CNN backbone extracts range-discriminative features independently of the Transformer’s global context, preventing the model from ignoring local texture degradation (fog) in favor of global chromatic biases. The classification loss $L_{cls}$ and auxiliary bin consistency loss $L_{bin}$ are defined as:

(11) $$L_{cls}=-\sum_{i=1}^{N}\sum_{k=1}^{K}y_{i,k}\log\left({\hat{p}}_{cls}^{\left(i,k\right)}\right)$$

(12) $$L_{bin}=-\sum_{i=1}^{N}\sum_{k=1}^{K}y_{i,k}\log\left({\hat{p}}_{bin}^{\left(i,k\right)}\right)$$

where $y_{i,k}$ is the ground-truth indicator (1 if sample *i* belongs to bin *k*, 0 otherwise), *K* is the number of bins, ${\hat{p}}_{cls}^{\left(i,k\right)}$ is the predicted probability for bin *k* from the classification head, and ${\hat{p}}_{bin}^{\left(i,k\right)}$ is the predicted probability from the bin consistency head. To enforce the physical consistency of the learnable thresholds, a regularization term $L_{thr}$ is introduced. This term penalizes the learnable thresholds τ if they deviate beyond the statistical distribution of the assigned visibility bins. This constraint ensures that the attention gates for the 0–10 km bin do not inadvertently focus on features only visible at 40 km, thereby maintaining alignment with the physical principles of atmospheric scattering.

(13) $$L_{thr}=\frac{1}{K}\sum_{k=1}^{K}\left[{\left(\tau_{c}^{\left(k\right)}-\tau_{co}^{\left(k\right)}\right)}^{2}+{\left(\tau_{s}^{\left(k\right)}-\tau_{so}^{\left(k\right)}\right)}^{2}\right]$$

where *K* is the total number of bins, $\tau_{c0}^{\left(k\right)}$ and $\tau_{s0}^{\left(k\right)}$ are the Koschmieder-derived initial values, while $\tau_{c}^{\left(k\right)}$ and $\;\tau_{s}^{\left(k\right)}$ are the learned thresholds for the *k*-th bin’s channel and spatial attention, respectively. These initial thresholds $\tau_{c0}^{\left(k\right)}$ and $\tau_{S0}^{\left(k\right)}$ are derived from the expected contrast attenuation at bin midpoints $V_{mid}^{\left(k\right)}$ based on Koschmieder’s law:

(14) $$\tau_{c0}^{\left(k\right)}=\tau_{S0}^{\left(k\right)}=\exp\left(-\frac{3.912}{V_{mid}^{\left(k\right)}}\right)$$

In this formulation, $V_{mid}^{\left(k\right)}$ corresponds to the representative distances of the five bins (i.e., 5 km, 15 km, 25 km, 35 km, and 45 km). This regularization term helps to prevent the model from converging on mathematically valid but physically impossible feature mappings. For example, without this constraint, an attention branch assigned to the 0–10 km bin might erroneously focus on the overall luminance of the sky to minimize the loss function. The short-range bin is no longer focusing on short-range objects by finding a statistical shortcut which causes the range-aware mechanism to fail. The optimization strategy employs differential learning rates: base parameters (CNN/ViT weights) are trained with a rate of ${1\times 10}^{-4}$, whereas the learnable thresholds *τ* utilize a higher rate of ${2\times 10}^{-3}$ to facilitate rapid adaptation to varying atmospheric conditions. This integrated approach enables comprehensive visibility estimation where continuous distance prediction is refined through discrete range awareness, physical consistency enforcement, and multi-scale feature integration, which represents a significant advancement over conventional single-objective frameworks.

### 2.8. Data and Equipment

**Figure 4:**
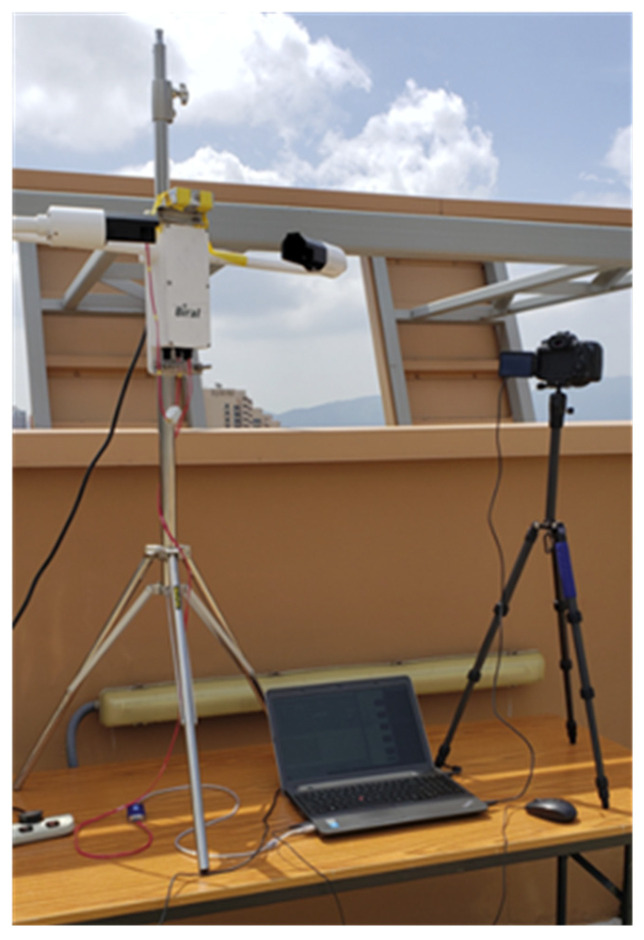
BIRAL SWS-100 Visibility Sensor.

Experiments were conducted on a Linux system equipped with an NVIDIA RTX 5090 GPU, with detailed specifications outlined in Table 2. A significant challenge in visibility estimation research is the scarcity of large-scale, publicly available datasets with high-quality, instrument-calibrated ground truth visibility values. To address this gap and support reproducible research, we contribute the Hong Kong Chu Hai College Visibility Dataset (HKCHC-VD), which comprises 11,148 images paired with corresponding certified visibility measurements. The Canon EOS 90D with an 18 mm focal length lens was used to capture images with a resolution of 6960 × 4640 pixels. The camera was aligned precisely with the Biral SWS-100 sensor to capture visibility values synchronously via a computer (see Figure 4). This sensor provides certified measurements across a 10 m to 75 km range, with accuracy validated against reference transmissometers. Data collection is performed from 8:00 to 18:00 daily for two months across diverse atmospheric conditions. Images with extreme lens flare, heavy rain, or physical obstructions were filtered out. Subsequently, the data was categorized into five visibility ranges (0–10 km, 10–20 km, 20–30 km, 30–40 km, 40–50 km) for evaluation. To reflect real-world meteorological conditions, the dataset exhibits an inherent distribution imbalance: a higher frequency of measurements was recorded in the 20–50 km range, while the 0–20 km range contains fewer samples. The dataset was partitioned into training (8921 images; 80%) and testing sets (2227 images; 20%), with the distribution across visibility ranges detailed in Table 3.

## 3. Results

This section outlines the experimental objectives, evaluation strategy, and sequence of experiments used to validate the proposed visibility estimation framework. The primary goal is to quantify both numerical accuracy (regression) and categorical reliability (bin classification) across diverse atmospheric conditions. The experimental results comprise controlled ablation studies, comparisons with established baselines, and qualitative inspections of attention maps to confirm the methodology of the proposed framework.

### 3.1. Hyperparameter Rationale and Stability

Visibility estimates from our algorithm were compared against ground-truth measurements from the Biral SWS-100 sensor. We employ Mean Absolute Error (MAE) and Mean Squared Error (MSE) as evaluation metrics:

(15) $$MAE=\frac{1}{N}\sum_{i=1}^{N}\left|v_{i}-{\hat{v}}_{i}\right|\;\;\;\;$$

(16) $$MSE=\frac{1}{N}\sum_{i=1}^{N}{\left(v_{i}-{\hat{v}}_{i}\right)}^{2}$$

where *N* is the total number of samples, $v_{i}$ is the measured visibility, and ${\hat{v}}_{i}$ is the estimated visibility for the *i*-th sample. The AdamW optimizer was employed with differential learning rates:

- Base parameters (CNN/ViT): 1 × 10^−4^
- Threshold parameters: 2 × 10^−3^

Training and validation data were processed in batches of 64, with shuffling enabled for the training set. Key hyperparameters are summarized in Table 4. The multi-task loss function uses the weighting scheme detailed in Table 5. The weights were determined through a grid search over the values {0.1, 0.2, 0.5, 1.0}. We observed that setting β > 0.5 led to the model achieving high classification accuracy but suffered from higher Mean Absolute Error (MAE) in regression. The chosen configuration (γ = 1.0, β = 0.2) represents the optimal balance between categorical boundary awareness and continuous numerical precision. A small regularization penalty λ = 0.01 is applied to ensure that the learnable thresholds τ do not deviate into physically impossible values, thereby maintaining alignment with Koschmieder’s law. By providing multiple supervision paths, the model achieves faster convergence and is less sensitive to the initialization of individual backbone weights.

### 3.2. Visibility Estimation and Classification Results

To ensure a rigorous evaluation of the proposed framework, we conducted a comprehensive performance analysis. This included an ablation study to validate the architectural components, a comparative analysis against established baselines, and a qualitative examination of the learned attention maps to verify physical consistency.

We evaluated multiple architectural configurations to isolate the contributions of dual-backbone design and the Range-Aware Learnable Threshold Attention. The baseline model ResNeXt-50 (baseline) [42] employed a single-backbone ResNeXt-50 with Convolutional Block Attention Module (CBAM) attention [41], processing images through a purely convolutional pathway. Building upon this foundation, we evaluated hybrid configurations to assess the impact of attention mechanisms and model architecture.

First, we tested the performance of single-branch ResNeXt-50 variants enhanced with range-aware attention. This mechanism introduced bin-specific attention modules and learnable thresholds τ to dynamically suppress irrelevant regions based on visibility conditions. Within this single-branch category, the ResNeXt-50 (spatial-threshold) variant applied learnable thresholds $\tau_{c}$ exclusively to spatial attention maps, while the ResNeXt-50 (dual-threshold) variant jointly refined spatial and channel attention mechanisms using both thresholds $\tau_{c}$ and $\tau_{s}$. Additionally, a ResNeXt-50 (no RAT-Attn) version was evaluated, which retained dual attention modules but omitted the thresholding operation entirely.

Second, we implemented hybrid ResNeXt-50 + ViT-B/16 architectures with three corresponding variants. Similarly, the ResNeXt-50 + ViT (spatial-threshold) model applied thresholds exclusively to spatial attention maps within the hybrid framework, whereas the ResNeXt-50 + ViT (dual-threshold) configuration jointly refined both attentions using $\tau_{c}$ and $\tau_{s}$. Finally, the ResNeXt-50 + ViT (no RAT-Attn) variant retained the dual attention modules without applying learnable thresholds.

Pre-trained weights from the ImageNet-1K dataset were used for both ResNeXt and ViT backbones, with architectural modifications to align with the regression task. Specifically, the ResNeXt backbone removed its final two layers (global pooling and classification head), while the ViT-B/16 backbone omitted its final classification head. This systematic ablation study enabled a precise assessment of how ViT integration, attention dimensions, and thresholding mechanisms jointly influence performance across diverse visibility conditions.

Low visibility (0 to 10 km) represents the most critical regime for transport safety, where dense fog and haze obscure distant cues. In this range, the CNN backbone plays a primary role with precise edge and texture detection of close objects. As shown in Table 6, our ResNeXt-50 architecture with dual-threshold demonstrated an improved capability in low-visibility scenarios, achieving a 47% reduction in MSE (4.48 km^2^) and 21% improvement in MAE (1.61 km) compared to the baseline. This confirms that the threshold parameter τ is effective for suppressing irrelevant noise channels in fog.

The mid-range visibility regime (10 to 30 km) serves as a transition zone where the CNN identifies intermediate landmarks while the ViT models atmospheric attenuation gradients. As detailed in Table 7, the ResNeXt-50 + ViT (spatial-threshold) variant proved effective for the 10 to 20 km range, achieving the lowest MAE (0.84 km) and the highest accuracy (93.33%). For the 20 to 30 km range, the ResNeXt-50 + ViT (no RAT-Attn) model shows competitive performance, which indicates that conventional thresholding can occasionally suppress subtle horizon features necessary for mid-range estimation.

In high-visibility conditions (30 to 50 km), the ViT backbone dominates horizon-line analysis, supplemented by CNN texture features. Table 8 shows that the ResNeXt-50 (dual-threshold) model reached higher performance in the 30 to 40 km bin with an MSE of 5.96 km^2^ and MAE of 1.9 km. Both single and hybrid backbones with learnable thresholds demonstrated competitive performance in extreme visibility (40 to 50 km), with the ResNeXt-50 + ViT (spatial-threshold) model achieving the lowest MAE (1.75 km) This indicates that thresholding is less critical in clear regimes.

A comprehensive analysis indicates that the ResNeXt-50 + ViT (spatial-threshold) architecture achieves the best overall performance (MAE: 1.62 km, MSE: 5.70 km^2^), while the ResNeXt-50 (dual-threshold) model offers the most balanced solution across all ranges. Crucially, the absence of thresholding caused notable degradation in low-visibility scenarios (MAE increased from 1.61 to 1.92 km for ResNeXt-50). Furthermore, the ResNeXt-50 + ViT (spatial-threshold) model achieved the highest overall bin accuracy (87.38%). This indicates that without the learnable threshold τ to filter atmospheric noise, the model struggles to distinguish between relevant features and visual artifacts in fog. Generally, ViT integration consistently improved performance in low visibility ranges (10 to 20 km), while thresholding mechanisms provided advantages in low-visibility conditions (0 to 10 km). The ResNeXt-50 + ViT (spatial-threshold) variant demonstrated the most consistent performance across all visibility ranges, highlighting the value of spatial attention refinement without channel thresholding.

**Figure 5:**
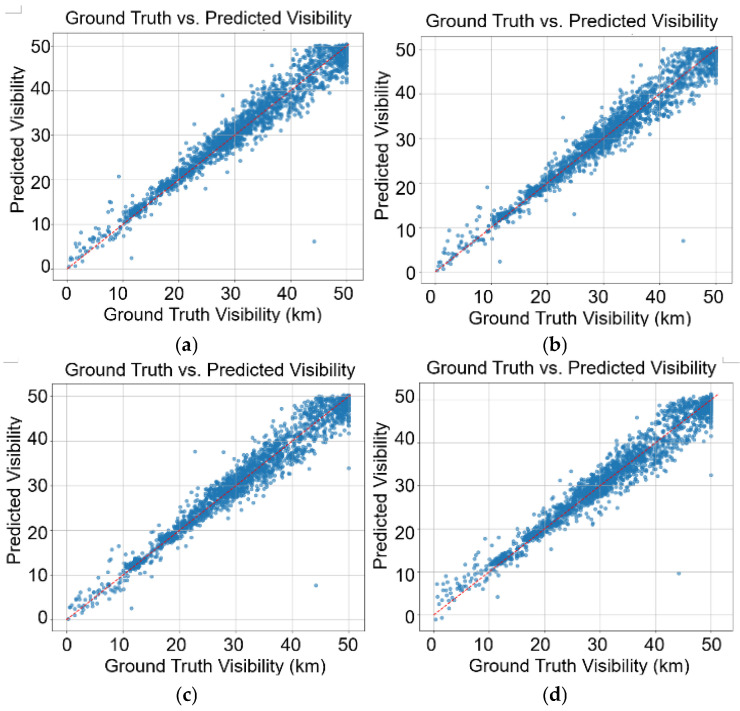
Ground Truth vs. Predicted Visibility. (**a**) ResNXet-50 only (baseline), (**b**) ResNeXt-50 +ViT (spatial-threshold), (**c**) ResNeXt-50 + ViT (dual-threshold), and (**d**) ResNeXt-50 + ViT (no RAT-Attn).

Figure 5 plots the ground truth against the predicted visibility for the ResNeXt-50 baseline, ResNeXt-50 + ViT (spatial-threshold), ResNeXt-50 + ViT (dual-threshold), and ResNeXt-50 + ViT (no RAT-Attn) models. A critical challenge noted was the scarcity of data in the 0–10 km range (239 training samples vs. 3087 for 40–50 km). Despite this severe imbalance, our multi-task loss function allowed the model to achieve a classification accuracy of 89.29% (Table 6) and high regression precision in this minority class. This indicates that the auxiliary bin consistency head effectively acts as a regularizer, preventing the model from overfitting to the majority class.

**Figure 6:**
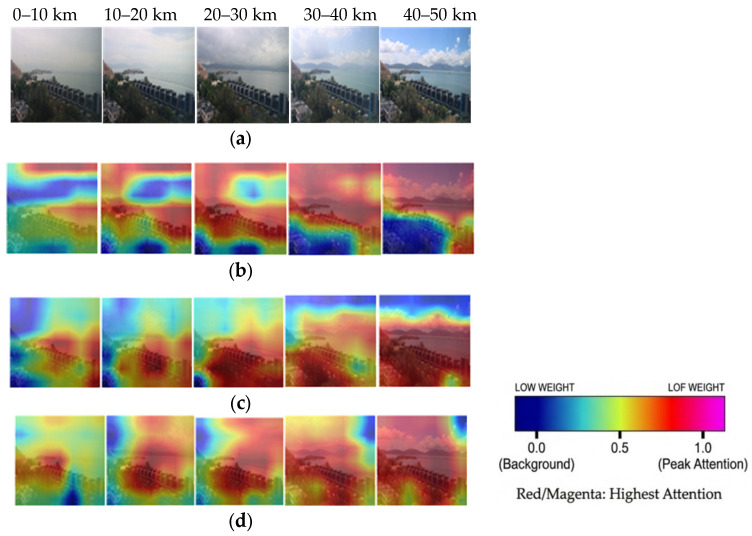
Visibility-range-aware Attention Heatmap. (**a**) Original image, (**b**) ResNeXt-50 (baseline), (**c**) ResNeXt-50 + ViT (spatial-threshold), and (**d**) ResNeXt-50 + ViT (no RAT-Attn).

Spatial channel attention heatmaps in Figure 6 illustrate how different models prioritize regions for visibility estimation across atmospheric conditions. Comparisons between the ResNeXt-50 (baseline), ResNeXt-50 + ViT (spatial-threshold), and ResNeXt-50 + ViT (no RAT-Attn) visualizations reveal that the spatial threshold architecture exhibits the most physically plausible attention distributions, dynamically adapting to visibility-specific atmospheric conditions.

For low-visibility scenarios (0–10 km), the spatial threshold model concentrates on near-field objects critical for fog analysis, while the baseline model erroneously prioritizes intermediate and sky regions with lower relevance. For mid-range visibility (10–30 km), the spatial threshold model highlights a broader set of features, balancing near-field objects and distant landmarks (e.g., sea, hills), whereas the baseline model overemphasizes close-range textures and atmospheric gradients. In high-visibility conditions (30–50 km), the spatial threshold model suppresses high-variance regions like clouds and sky while maintaining awareness of the overall scene context. Conversely, the baseline model disproportionately highlights distant objects while suppressing critical near-field references. These patterns validate the threshold mechanism’s ability to dynamically reconfigure attention maps, precisely suppress features in noisy regions, and contextually balance near, mid, and far elements according to atmospheric conditions.

In contrast, the baseline model (Figure 6b) erroneously prioritizes sky regions in low visibility, leading to poor generalization. This adaptive behavior confirms that the RAT-Attn module successfully learns to replicate the human observer’s “instinctive” focus, prioritizing regions based on atmospheric transparency.

### 3.3. Analysis of Computational Complexity

The analysis of model complexity and inference speed is shown in Table 9. By comparing the ResNeXt-50 (baseline) with the single-branch ResNeXt-50 with RAT-Attn, we observe that the introduction of the Range-Aware Attention mechanism adds a minimal computational footprint. The parameter increases by 2.39 M (~9.6%), primarily due to the addition of the bin predictor and the parallel attention heads. Notably, the GFLOPs remain constant at 4.29, indicating that the learnable thresholding and gating operations are mathematically lightweight relative to the convolutional backbone. The marginal increase in latency from 2.08 to 2.60 ms demonstrates that our range-aware optimization provides significant domain-specific refinement without compromising the model’s high-speed throughput.

The hybrid ResNeXt-50 + ViT-B/16 with RAT-Attn is the most complex configuration in our approach, with 113.93 M parameters and 15.58 GFLOPs. This nearly fourfold increase in computational complexity is a direct result of the Vision Transformer (ViT-B/16) backbone’s global self-attention mechanisms. However, when deployed on the NVIDIA RTX 5090, the inference latency only rises to 5.53 ms. This suggests that while the hybrid model is significantly heavier in terms of raw math, the modern GPU’s massive parallel processing capabilities and high memory bandwidth effectively mitigate the overhead. Even in hybrid ResNeXt-50 + ViT-B/16 with RAT-Attn is the most complex form, the model maintains an inference speed of over 180 FPS. In the context of meteorological monitoring where visibility measurements are typically updated at intervals of 1 to 10 min, a latency of 5.53 ms is exceptionally efficient. These results confirm that the proposed RAT-Attn framework is well-suited for high-precision, real-time environmental monitoring in safety-critical transportation sectors.

While the hybrid ResNeXt-50 and ViT-B/16 architecture provides notable accuracy, its 113.93 M parameter count poses challenges for devices with limited VRAM and computational power, such as the NVIDIA Jetson series or embedded ARM-based processors. The single-branch ResNeXt-50 with RAT-Attn is actually the good candidate for edge deployment. It strikes a balance between the depth needed for meteorological physics and the efficiency required for embedded hardware.

### 3.4. Impact of the Sharpness Factor

To evaluate the influence of the attention gate transition on visibility estimation, we conducted an ablation study on the sharpness factor α defined in Equations (4) and (7). We tested α∈ {5, 10, 15, 20} to observe how the thresholding should be used for optimal feature extraction.

**Figure 7:**
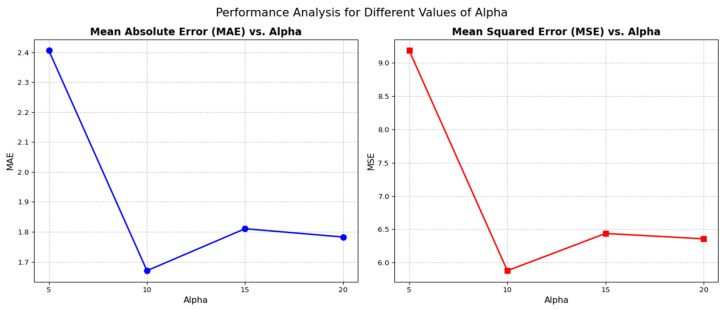
Performance Analysis for Different Values of Alpha.

As shown in Figure 7, the ResNeXt-50 + ViT (dual-threshold) model’s overall precision is highly sensitive to the sharpness of the attention transition. At α = 5, the model produces its highest errors (MAE = 2.41 km, MSE = 9.2 km^2^). The attention transition is too gradual, failing to effectively suppress irrelevant features or clearly isolate distance-specific atmospheric cues. The optimal performance is achieved at α = 10, where both MAE and MSE reach their global minima (1.67 km and 5.88 km^2^, respectively). As α increases to 15 and 20, the error slightly rises. This indicates that excessive sharpness may lead to information loss, as some subtle but relevant gradient cues are prematurely masked, resulting in a decrease in regression head accuracy.

**Figure 8:**
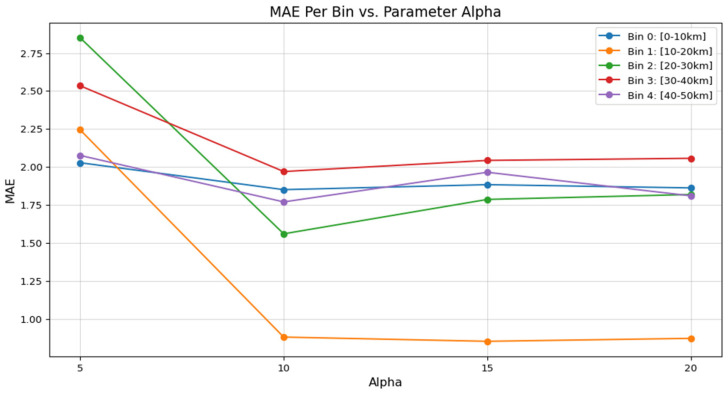
Comparison of Sharpness Factor Across Different Visibility Ranges.

Figure 8 illustrates how the sharpness factor affects different visibility ranges. The mid-range visibility bins (Bin 1: 10–20 km and Bin 2: 20–30 km) show the most dramatic improvement when moving from α = 5 to α = 10. Specifically, for Bin 2, the MAE drops from nearly 2.85 km to 1.56 km. This indicates that mid-range visibility estimation relies heavily on the model’s ability to sharply define the boundaries of distant structural features against moderate haze. Conversely, the low visibility range (0–10 km) remains relatively stable across all α values (see the blue line). This indicates that visual degradation is so pervasive in dense fog that the sharpness of the attention gate is less important than the overall detection of global brightness and contrast loss. However, for clear weather conditions (40–50 km), the model benefits from a balanced sharpness of α = 10, as it requires a precise threshold to identify low-contrast features at very far distances.

## 4. Discussion

We compared our proposed approach against VisNet [17], a landmark ANN-based visibility estimation method [43], and a stronger backbone configuration utilizing NFNet-F0. Because the original method described in [17] did not provide continuous visibility values, it was augmented with the same regression head used in our model to facilitate a fair comparison. Furthermore, as the framework in [43] achieved its best performance using ResNet-50 [14] for feature extraction, we conducted a subrange visibility analysis across all approaches using ResNet-50 as the common feature extractor.

Table 10 presents the estimated results for low-visibility (0 to 10 km) and mid-visibility conditions (10 to 30 km), while Table 11 displays for high-visibility (30 to 50 km) conditions. As shown in Table 10 and Table 11, the modified VisNet generally struggles in extreme visibility ranges. In the critical 0 to 10 km range, VisNet recorded an MSE of 19.01 km^2^ and MAE of 2.35 km, significantly lagging behind the proposed attention-based models. While it shown competitive stability in the 10 to 20 km range (MSE: 2.24 km^2^), its overall performance (MSE:6.91 km^2^, MAE:1.77 km) indicates that without range-aware mechanisms, standard convolutional streams struggle to capture the non-linear optical degradation present in highly foggy or very clear atmospheric conditions.

In critical low-visibility conditions (0 to 10 km), the ResNet-50 + ViT (dual-threshold) model achieves the best regression performance (MSE: 6.06 km^2^, MAE: 1.71 km), while the method in [43] leads in classification accuracy (95%). During mid-visibility conditions (20–30 km), the ResNet-50 + ViT (spatial-threshold) model excels in both regression (MSE: 3.82 km^2^, MAE: 1.46 km) and classification (82.12%). For higher visibility (30 to 50 km), the ResNet-50 + ViT (no RAT-Attn) variant shows competitive regression at 30 to 40 km (lowest MSE: 6.37 km^2^), and the spatial-threshold model leads classification at 40 to 50 km (93.1%).

The ResNet-50 + ViT (spatial-threshold) model delivers the strongest overall results, achieving the lowest combined MSE (5.87 km^2^) and MAE (1.65 km), alongside the highest overall classification accuracy (87.07%). Notably, all new models demonstrate substantial improvements over the approach in [17,43] for low-visibility regression (e.g., >75% lower MSE at 0 to 10 km) and mid-visibility performance. While the approach in [43] retains a marginal advantage in high-visibility regression (40 to 50 km), the method in [17] fails to demonstrate performance advantages across low, mid, or high visibility conditions. These show that the spatial-threshold model offers the most balanced performance, while the dual-threshold model is particularly effective for low-visibility regression tasks.

To test robustness across architectures, we implemented the framework using the NFNet-F0 backbone [44], which produces larger feature dimensions (3072-D) compared to ResNeXt-50 (2048-D). The results in Table 12 confirm that the advantage of the dual-threshold approach is architecture-agnostic. In the critical low-visibility conditions (0–10 km), the dual-threshold NFNet model achieved an MSE of 5.19 km^2^ and MAE of 1.50 km, outperforming both the spatial-threshold (MSE: 6.84 km^2^, MAE: 1.68 km) and no RAT-Attn (MSE: 9.39 km^2^, MAE: 1.95 km) variants. This represents improvements of 24.1% and 44.7% in MSE, and 10.7% and 23.1% in MAE, respectively. This consistency across different backbones (ResNeXt, ResNet, NFNet) validates that the performance gains are statistically meaningful and not attributable to random noise.

## 5. Conclusions

This paper proposes a Range-Aware Attention mechanism that applies bin-specific learnable thresholds to both channel and spatial attention operations. Simultaneously, the multi-head prediction architecture establishes mutually reinforcing optimization through complementary supervision streams from the regression head, classification head, and auxiliary bin consistency heads. This integrated design assists with adaptive feature processing tailored to diverse visibility conditions, which dynamically suppresses noise while enhancing visibility-critical features across various atmospheric conditions. Our experimental studies demonstrate that the ResNeXt-50 + ViT (spatial-threshold) architecture achieves the most consistent overall performance, with an MAE of 1.62 km and MSE of 5.70 km^2^. This configuration shows an improvement over the ResNeXt-50 (baseline) by 3.4% in MSE and 4.7% in MAE across the entire 0 to 50 km visibility range. Notably, ablation studies indicate that the absence of thresholding results in notable performance degradation in low-visibility scenarios, which validates the utility of our adaptive attention mechanism for robust visibility estimation.

## Figures and Tables

**Table 1:** Performance of State-of-the-art VLMs in visibility estimation.

Ground Truth	Estimated Visibility Value (km) (Prompting Strategy: Estimate the Meteorological Visibility in Kilometers for This Image. Provide a Single Numerical Value or a Narrow Range)			
	GPT-5 Mini	Qwen3-VL (235B)	Grok-4	Gemini-2.5-Pro
7.6 km	0.5–1	0.3–0.5	0.5	1–2
14.9 km	1–2	1–1.5	2	2–4
27.5 km	2–4	2–2.5	5	5–8
30.5 km	5–8	5–6	10	10–20
49.6 km	15–25	18–20	25	>30

**Table 2:** Hardware Configuration.

Item	Configuration
Operating System	Linux
Memory Capacity	64 GB
Central Processing Unit	AMD Ryzen 9 9950X 16-Core Processor
Graphical Processing Unit	NVIDIA GeForce RTX 5090

**Table 3:** HKCHC Visibility Dataset.

	Visibility Range (km)				
	0–10	10–20	20–30	30–40	40–50
No. of Training Sample Images	239	1141	2051	2403	3087
No. of Test Sample Images	59	285	512	600	771
Total:	298	1426	2563	3003	3585

**Table 4:** Training Hyperparameters.

Hyperparameters	Value or Setting
Optimizer	AdamW
Base LR	1 × 10^−4^
Threshold LR	2 × 10^−3^
Batch Size	64
Shuffle	Enabled (train)/Disabled (val)

**Table 5:** Loss weighting.

Component	Weight
Regression Loss ($L_{reg}$)	γ = 1.0
Classification Loss ($L_{cls}$)	β_1_ = 0.2
Bin Consistency ($L_{bin}$)	β_2_ = 0.2
Threshold Reg ($L_{thr}$)	Λ = 0.01

**Table 6:** Results for Low-Visibility Conditions.

Visibility Range		0–10 km	
Performance Evaluation Index		MSE	MAE
ResNeXt-50 (baseline)	Prediction Errors	8.49	2.03
	Classification Accuracy	NA	
ResNeXt-50 (spatial-threshold)	Prediction Errors	7.37	1.92
	Classification Accuracy	89.29%	
ResNeXt-50 (dual-threshold)	Prediction Errors	4.48	1.61
	Classification Accuracy	87.5%	
ResNeXt-50 (no RAT-Attn)	Prediction Errors	7.43	1.92
	Classification Accuracy	78.57%	
ResNeXt-50 + ViT (spatial-threshold)	Prediction Errors	7.23	1.73
	Classification Accuracy	86.44%	
ResNeXt-50 + ViT (dual-threshold)	Prediction Errors	7.09	1.85
	Classification Accuracy	82.14%	
ResNeXt-50 + ViT (no RAT-Attn)	Prediction Errors	9.23	2.04
	Classification Accuracy	78.57%	

**Table 7:** Result for Mid-Visibility Conditions.

Visibility Range		10–20 km		20–30 km	
Performance Evaluation Index		MSE	MAE	MSE	MAE
ResNeXt-50 (baseline)	Prediction Errors	1.98	1.08	4.1	1.51
	Classification Accuracy	NA		NA	
ResNeXt-50 (spatial-threshold)	Prediction Errors	1.62	0.87	4.12	1.56
	Classification Accuracy	90.43%		74.07%	
ResNeXt-50 (dual-threshold)	Prediction Errors	1.57	0.9	4.72	1.66
	Classification Accuracy	92.91%		76.42%	
ResNeXt-50 (no RAT-Attn)	Prediction Errors	1.98	0.94	3.84	1.48
	Classification Accuracy	91.13%		75.44%	
ResNeXt-50 + ViT (spatial-threshold)	Prediction Errors	1.48	0.84	4.1	1.5
	Classification Accuracy	93.33%		84.57%	
ResNeXt-50 + ViT (dual-threshold)	Prediction Errors	1.6	0.88	4.36	1.56
	Classification Accuracy	92.55%		79.37%	
ResNeXt-50 + ViT (no RAT-Attn)	Prediction Errors	1.91	1.0	3.42	1.36
	Classification Accuracy	91.84%		90.77%	

**Table 8:** Results for High-Visibility Conditions.

Visibility Range		30–40 km		40–50 km		Overall	
Performance Evaluation Index		MSE	MAE	MSE	MAE	MSE	MAE
ResNeXt-50 (baseline)	Prediction Errors	6.12	1.95	8.04	1.79	5.9	1.7
	Classification Accuracy	NA		NA		NA	
ResNeXt-50 (spatial-threshold)	Prediction Errors	6.18	1.96	7.89	1.78	5.75	1.67
	Classification Accuracy	84.42%		91.93%		85.53%	
ResNeXt-50 (dual-threshold)	Prediction Errors	5.96	1.9	7.89	1.76	5.75	1.66
	Classification Accuracy	85.43%		90.49%		86.12%	
ResNeXt-50 (no RAT-Attn)	Prediction Errors	6.37	2.03	8.24	1.9	5.9	1.72
	Classification Accuracy	85.43%		90.89%		85.58%	
ResNeXt-50 + ViT (spatial-threshold)	Prediction Errors	6.12	1.95	7.89	1.75	5.7	1.62
	Classification Accuracy	80.63%		92.35%		87.38%	
ResNeXt-50 + ViT (dual-threshold)	Prediction Errors	6.33	1.97	8.01	1.77	5.88	1.67
	Classification Accuracy	82.58%		92.19%		86.44%	
ResNeXt-50 + ViT (no RAT-Attn)	Prediction Errors	6.78	2.03	8.68	1.8	6.11	1.66
	Classification Accuracy	75.38%		88.8%		85.76%	

**Table 9:** Comparison of Model Complexity and Inference Speed.

Framework	Params (M)	FLOPs (G)	Latency (ms)
ResNeXt-50 (baseline)	24.82 M	4.29	2.08
ResNeXt-50 (dual-threshold)	27.21 M	4.29	2.60
ResNeXt-50 + ViT (dual-threshold)	113.93 M	15.58	5.53

**Table 10:** Comparison of Visibility Estimation and Classification for Low/Mid-Visibility Conditions.

Visibility Range		0–10 km		10–20 km		20–30 km	
Performance Evaluation Index		MSE	MAE	MSE	MAE	MSE	MAE
VisNet [17] + Regression head	Prediction Errors	19.01	2.35	2.24	1.01	5.76	1.75
	Classification Accuracy	NA		NA		NA	
Landmark ANN-based Method [43]	Prediction Errors	25.5	1.84	2.24	0.87	5.46	1.66
	Classification Accuracy	95%		90%		85%	
ResNet-50 + ViT (spatial-threshold)	Prediction Errors	6.25	1.77	1.52	0.91	3.82	1.46
	Classification Accuracy	85.71%		90.78%		82.12%	
ResNet-50 + ViT (dual-threshold)	Prediction Errors	6.06	1.71	1.63	0.91	4.43	1.58
	Classification Accuracy	89.29%		92.55%		78.19%	
ResNet-50 + ViT (no RAT-Attn)	Prediction Errors	10.3	2.05	1.61	0.87	4.19	1.47
	Classification Accuracy	80.36%		90.43%		79.76%	

**Table 11:** Comparison of Visibility Estimation and Classification for High-Visibility Conditions.

Visibility Range		30–40 km		40–50 km		Overall	
Performance Evaluation Index		MSE	MAE	MSE	MAE	MSE	MAE
VisNet [17] + Regression head	Prediction Errors	7.96	2.14	7.65	1.73	6.91	1.77
	Classification Accuracy	NA		NA		NA	
Landmark ANN-based Method [43]	Prediction Errors	7.66	2.15	5.91	1.69	6.38	1.71
	Classification Accuracy	81%		91%		86.9%	
ResNet-50 + ViT (spatial-threshold)	Prediction Errors	7.02	2.03	7.91	1.75	5.87	1.65
	Classification Accuracy	81.91%		93.1%		87.07%	
ResNet-50 + ViT (dual-threshold)	Prediction Errors	6.65	1.95	8.47	1.86	6.12	1.69
	Classification Accuracy	83.08%		91.93%		86.39%	
ResNet-50 + ViT (no RAT-Attn)	Prediction Errors	6.37	2.01	8.45	2.07	6.08	1.76
	Classification Accuracy	82.24%		92.97%		86.39%	

**Table 12:** Performance Evaluation of NFNet-F0 + ViT Model.

Visibility Range (km)	Performance Metric	NFNet-F0 + ViT		
		Spatial-Threshold	Dual-Threshold	No RAT-Attn
0–10	MSE (km^2^)	6.84	5.19	9.39
	MAE (km)	1.68	1.5	1.95
	Accuracy	91.53%	91.53%	84.75%
10–20	MSE (km^2^)	1.51	1.49	2.03
	MAE (km)	0.83	0.82	1.0
	Accuracy	89.47%	91.93%	84.21%
20–30	MSE (km^2^)	3.93	3.55	3.74
	MAE (km)	1.48	1.43	1.45
	Accuracy	83.01%	83.79%	85.35%
30–40	MSE (km^2^)	6.01	5.89	6.88
	MAE (km)	1.93	1.89	2.05
	Accuracy	82.94%	85%	83.17%
40–50	MSE (km^2^)	7.97	8.03	8.05
	MAE (km)	1.78	1.81	1.82
	Accuracy	91.57%	90.79%	91.96%
Overall	MSE (km^2^)	5.66	5.51	6.01
	MAE (km)	1.63	1.61	1.69
	Accuracy	87.02%	87.79%	86.89%

## Data Availability

Data are contained within the article.
